# Plant Transcriptome Reprograming and Bacterial Extracellular Metabolites Underlying Tomato Drought Resistance Triggered by a Beneficial Soil Bacteria

**DOI:** 10.3390/metabo11060369

**Published:** 2021-06-09

**Authors:** Rafael J. L. Morcillo, Juan I. Vílchez, Song Zhang, Richa Kaushal, Danxia He, Hailing Zi, Renyi Liu, Karsten Niehaus, Avtar K. Handa, Huiming Zhang

**Affiliations:** 1 Shanghai Center for Plant Stress Biology, Center for Excellence in Molecular Plant Sciences, Chinese Academy of Sciences, Shanghai 201602, China; rafael@sibs.ac.cn (R.J.L.M.); juan@sibs.ac.cn (J.I.V.); zhangsong5861927@sdau.edu.cn (S.Z.); richa@sibs.ac.cn (R.K.); dxhe@sibs.ac.cn (D.H.); zihailing@novogene.com (H.Z.); ryliu@fafu.edu.cn (R.L.); 2 Institute for Water Research and Department of Microbiology, University of Granada, 18003 Granada, Spain; 3 Proteom-und Metabolomforschung, Fakultät für Biologie, Centrum für Biotechnologie, Universität Bielefeld, 33501 Bielefeld, Germany; kniehaus@CeBiTec.Uni-Bielefeld.DE; 4 Department of Horticulture and Landscape Architecture, Purdue University, West Lafayette, IN 47907, USA; ahanda@purdue.edu

**Keywords:** PGPR, tomato, drought stress, *Bacillus megaterium* TG1-E1, transcriptome, extracellular metabolites, osmoprotectant

## Abstract

Water deficit is one of the major constraints to crop production and food security worldwide. Some plant growth-promoting rhizobacteria (PGPR) strains are capable of increasing plant drought resistance. Knowledge about the mechanisms underlying bacteria-induced plant drought resistance is important for PGPR applications in agriculture. In this study, we show the drought stress-mitigating effects on tomato plants by the *Bacillus megaterium* strain TG1-E1, followed by the profiling of plant transcriptomic responses to TG1-E1 and the profiling of bacterial extracellular metabolites. Comparison between the transcriptomes of drought-stressed plants with and without TG1-E1 inoculation revealed bacteria-induced transcriptome reprograming, with highlights on differentially expressed genes belonging to the functional categories including transcription factors, signal transduction, and cell wall biogenesis and organization. Mass spectrometry-based analysis identified over 40 bacterial extracellular metabolites, including several important regulators or osmoprotectant precursors for increasing plant drought resistance. These results demonstrate the importance of plant transcriptional regulation and bacterial metabolites in PGPR-induced plant drought resistance.

## 1. Introduction

Drought stress, caused by water deficit, limits the worldwide utilization of arable lands as well as crop productivity [1]. In response to drought stress, plants undergo hyperosmotic signal transduction, leading to transcriptional reprograming for the repair of stress-induced damage, the re-balancing of cellular homeostasis, and the control of growth to adapt to the water deficit condition [2].

Plants live naturally with many microorganisms including plant growth-promoting rhizobacteria (PGPR), which are beneficial soil microorganisms that can stimulate plant growth and/or increase plant resistance to various stress conditions including drought stress [3,4,5]. Each PGPR strain produces a complex array of extracellular metabolites, some of which are responsible for triggering beneficial effects in plants. For instance, many PGPR strains exude the enzyme ACC (1-aminocyclopropane-1-carboxylate) deaminase that reduces plant ethylene levels, as well as siderophores that facilitate root uptake of metal nutrients [3]. Meanwhile, bacterial exopolysaccharides improve soil aggregation and maintain soil moisture in the rhizosphere and thus can help plants survive under water deficit conditions [6].

In response to PGPR, transcriptional reprograming in plant cells plays an important role in transducing the bacterial stimuli to the enhanced plant growth and stress resistance. For instance, *Bacillus amyloliquefaciens* GB03 enhances Arabidopsis root iron uptake, which is supported by the transcriptional upregulation of the root Fe^3+^ reductase *FRO2* and the Fe^2+^ transporter *IRT1*, as well as by the gene induction of *FIT1*, a transcription activator that controls *FRO2* and *IRT1* gene expression [7]. Likewise, a group of plant immunity-related genes were repressed by the bacterial volatile compound diacetyl, thereby supporting the function of diacetyl in establishing a beneficial association between PGPR and plants through suppression of immune responses in phosphate-sufficient plants [8].

While PGPR provide potentially powerful tools to improve plant stress resistance in agriculture, understanding the underlying mechanisms is challenging yet crucial for successful applications. In this study, we investigated whether and how *Bacillus megaterium* TG1-E1, a PGPR strain isolated from a high salinity environment [9], may affect drought stress resistance in tomato plants. The drought stress-alleviating effects of TG1-E1 on two different cultivars are demonstrated, followed by the analyses of plant transcriptome and bacterial extracellular metabolites, which indicate an integrated mechanism mediated through multiple key biological processes in plants as well as bacterial osmoprotectants.

## 2. Results and Discussion

### 2.1. Bacillus Megaterium TG1-E1 Increases Drought Resistance in Tomato Seedlings

**Figure 1:**
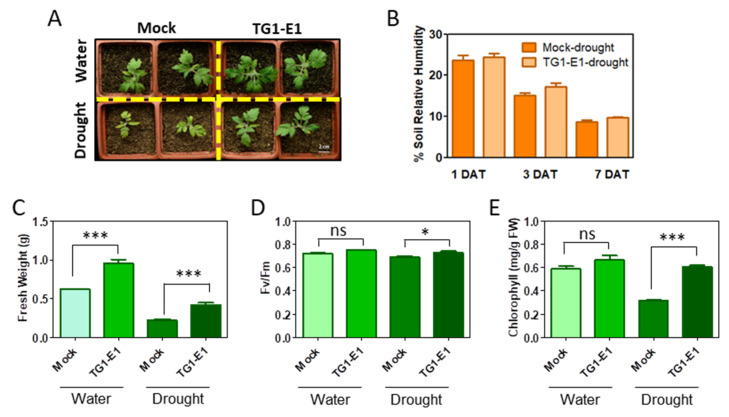
*Bacillus megaterium* TG1-E1 increases drought resistance in tomato seedlings. (**A**) TG1-E1 increased drought resistance in tomato (cultivar Micro-Tom) plants. Images were taken at 10 days after the drought treatment (DAT). (**B**) Relative humidity of the soil where tomato seedlings were grown. (**C**) Measurements of plant fresh weight. (**D**) Quantification of photosynthesis efficiency (Fv/Fm). (**E**) Measurements of chlorophyll contents. Results in panels (**C**–**E**) were from plants harvested at 10 DAT. The bar graphs show representative results from three independent experiments. Mean ± SE (*n* = 6 biological replicates). Asterisks denote significant differences at *p* < 0.05, Tukey’s multiple comparison test (*, *** and ns denote *p* < 0.05; *p* < 0.001 and *p* > 0.05, respectively).

To evaluate the effects of TG1-E1 on tomato drought resistance, we studied two cultivars of tomato, Micro-Tom and Ailsa Craig, with and without bacteria inoculation. At 10 days after the drought treatment started, the drought-treated Micro-Tom plants without TG1-E1 inoculation clearly suffered from water deficit, as evidenced by the arrested growth and leaf yellowing (Figure 1A). In contrast, the drought-treated plants with TG1-E1 inoculation showed more robust growth compared to their non-inoculated counterparts (Figure 1A), demonstrating the capacity of TG1-E1 in enhancing plant drought resistance in tomato. During the period of drought treatment, soil humidity decreased similarly in the soil both with and without TG1-E1 inoculation (Figure 1B), indicating that the increased plant drought resistance was unlikely due to bacteria-dependent water retention in the soil. Under the drought conditions, inoculation with TG1-E1 increased the Micro-Tom plant’s fresh weight by almost 50% (Figure 1C). In addition, plant photosynthesis efficiency and chlorophyll contents were higher in drought-stressed plants with TG1-E1 inoculation than in those without inoculation (Figure 1D,E). Similar results were observed with the Ailsa Craig plants (Figure S1), confirming the drought stress-alleviating effects of TG1-E1 on tomato plants. The plant-beneficial effects were supported by the successful colonization of TG1-E1 to tomato roots under both mock and drought conditions (Figure S2).

### 2.2. Bacillus Megaterium TG1-E1 Induces Transcriptomic Reprograming of in Tomato Plants under Drought Stress

#### 2.2.1. The Overall Functional Categorization of Differentially Expressed Genes (DEGs)

To gain insights into the mechanism for TG1-E1-increased plant drought resistance, we compared the transcriptomes between drought-stressed Micro-Tom with and without TG1-E1 inoculation. While the plants with and without TG1-E1-inoculation showed clearly contrasting phenotypes at 10 days after the drought treatment started (DAT), samples for the transcriptome analysis were harvested at 7 DAT, in order to catch the transcriptional regulation that is responsible for the downstream phenotypic changes. A total of 429 differentially expressed genes (DEGs) (Fold change ≥ 2; *p*-values ≤ 0.05) were identified, including 250 up-regulated DEGs and 179 down-regulated DEGs (Table S1).

**Figure 2:**
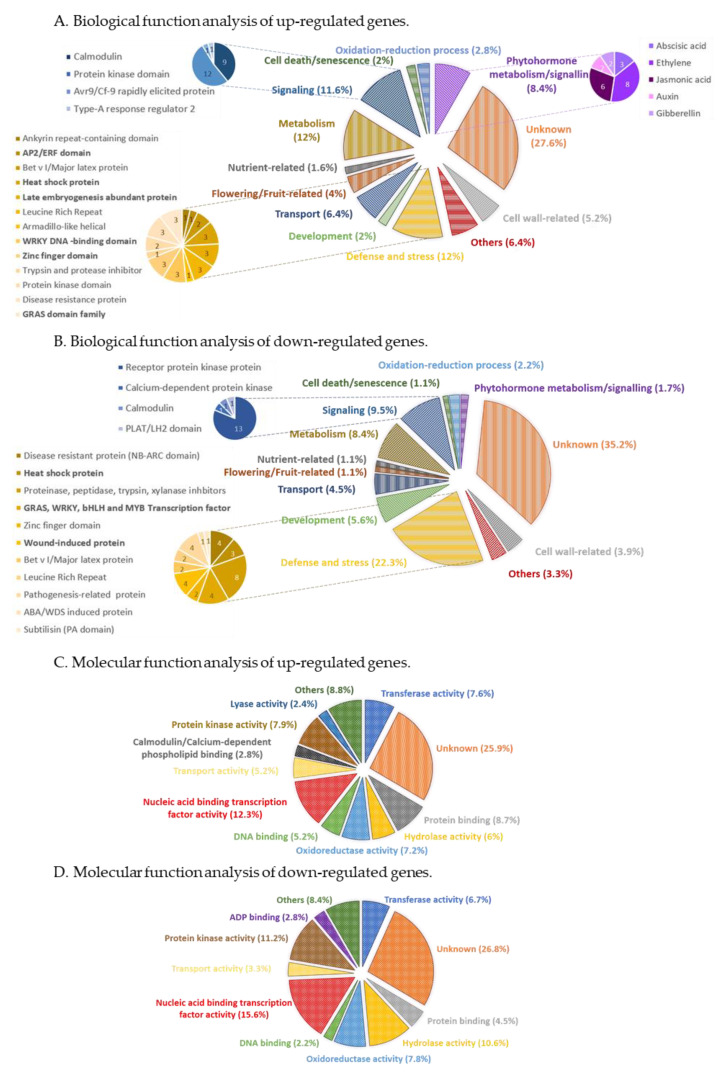
Functional categorization of tomato DEGs. Differentially expressed genes (DEGs) were identified by the comparison between drought-stressed plants with and without TG1-E1 inoculation. (**A**,**B**), Up- and down-regulated genes, respectively, are categorized based on their biological function. (**C**,**D**), Up- and down-regulated genes, respectively, are categorized based on their molecular function. Functional categorization was performed by using AgriGOv2. Numbers inside the diagram mean the amount of genes involved in each category.

To obtain an overall view of TG1-E1′s impacts on plant cellular functions, the TG1-E1-regualted DEGs were subjected to functional categorization by using AgriGOv2 (Figure 2 and Figure S3). Major biological functional categories of the up-regulated DEGs include unknown (27.6%), metabolism (12%), defense and stress (12%), signaling (11.6%) and phytohormone metabolism and signaling (8.6%) (Figure 2A). Meanwhile, main categories of down-regulated DEGs include unknown (35.2%), defense and stress (22.3%), signaling (9.5%) and metabolism (8.4%) (Figure 2B). Notably, the category of defense and stress accounts for 12% of up-regulated DEGs and 22% of down-regulated DEGs, whereas DEGs in this category are composed of genes encoding proteins with various functions (Figure 1A,B), indicating a complex interaction between plant responses to the biotic stimulus (TG1-E1) and the abiotic stressor (drought). In addition, the category of cell wall biosynthesis and organization was identified in both the up- and down-regulated DEGs (Figure 1A,B), indicating that TG1-E1-triggered plant drought resistance involves alterations in cell wall.

In order to identify molecular factors that potentially mediate TG1-E1-triggered plant drought resistance, we next performed DEG categorization by molecular function analysis. The results highlighted transcription factors and protein kinases among other categories, because these two categories are both abundant in DEGs which are either up- or down-regulated by TG1-E1 (Figure 2C,D).

#### 2.2.2. DEGs Implicating Regulation Mediated through Transcription Factors

Transcription factors are key regulators of plant responses to abiotic stress conditions including drought [10]. Genes encoding for transcription factors (TFs) accounted for 11% of all identified DEGs (Table 1), indicating that the observed transcriptional regulation by TG1-E1 would lead to a broadened impact on the plant transcriptome later on. Therefore, the enrichment of TFs in the DEGs demonstrates a key role of transcriptional regulation in mediating TG1-E1-triggered plant drought resistance.

Among the 48 TF DEGs are 22 zinc-finger family members (Table 1), some of which have been reported as responsive to hyperosmotic stress conditions such as drought and high salinity. For example, *SlWRKY75* and *SlWRKY45*, which are known to be repressed by drought stress [11,12], were further down-regulated by TG1-E1 in plants under the drought stress condition; meanwhile, TG1-E1 up-regulated *SlWRKY53* that was reported to be highly induced by salt stress [11]. Because TG1-E1 enhances plant resistance to drought stress, the TG1-E1-dependent transcriptional enhancement (either down- or up-regulation) highlights the function of these TFs in mediating plant adaptive responses to drought stress.

In addition to the zinc-finger TFs, DEGs encoding for TFs also include 10 APETALA2/ethylene-responsive factor (AP2/ERF) domain family members with eight DEGs being up-regulated, four GRAS family members with 3 DEGs being up-regulated, and seven MADS-box TFs which were all up-regulated by TG1-E1 (Table 1). These results strongly indicate that the amplification of transcriptional regulation is crucial for achieving TG1-E1-triggered plant drought resistance. The GRAS TFs play a significant role in tomato plant growth and development [13], while MADS-box TFs are key regulators of developmental plasticity in different plant species [14]. Therefore, the transcriptional regulation of these TFs provides clues to understanding how TG1-E1-treated plants adjust growth and development to adapt to the water deficit condition.

#### 2.2.3. DEGs Implicating Regulation of Drought-Responsive Signaling

Protein kinases, such as calmodulin-dependent protein kinases (CDPKs), mitogen-activated protein kinases (MAPKs) and receptor protein kinases (RPKs), are important regulatory components of plant signal transduction in response to various biotic and abiotic stress conditions. In the drought-stressed plants, TG1-E1 differentially regulated a total of 38 genes encoding for several types of protein kinases (Table 2). Notably, these DEGs are dominated by genes encoding for RPKs, with 14 and 16 RPKs up-regulated and down-regulated by TG1-E1, respectively (Table 2). The enrichment of RPKs in the kinase DEGs suggests that RPKs are important regulators of TG1-E1-triggered plant drought resistance. Meanwhile, four MAPKs (*SlMAPK3*, *SlMAPKKK14*, *SlMAPKKK21*, *SlMAPKKK59*), which have been implicated in tomato responses to environmental stress including drought [15], were up-regulated by TG1-E1 (Table 2), suggesting the important roles of these stress-response regulators in mediating TG1-E1-triggered plant drought resistance.

Calmodulins receive and transduce Ca^2+^ signals elicited by various stressors. One of the primary responses to drought stress is an increase in the cytosolic Ca^2+^ concentration and subsequent transduction of Ca^2+^ signals that promotes appropriate cellular responses in an effort to mitigate potential damages [2,16]. In the drought-stressed tomato, TG1-E1 increased gene expression of seven calmodulins and calmodulin-binding protein-like (CLMs) (Table 2). Notably, the CLM-encoding DEGs include *SlCBP60* (*Solyc03g119250*), which is orthologous to *AtSlCBP60g*, which positively regulates plant disease resistance and drought resistance in *Arabidopsis thaliana* [17]. In addition, TG1-E1 also up-regulated gene expression of two calcium-dependent lipid-binding proteins and two plasma-membrane calcium-transporting ATPases (*Solyc02g092450*; *Solyc02g064680*) (Table 2), which are key regulators of stress-induced calcium transients in plants [18,19,20]. Together these results indicate that bacterial modulation of plant Ca^2+^ signaling plays an important role in mediating TG1-E1-triggered drought resistance in tomato.

TG-E1 also induced gene expression of two root C4-dicarboxylate transporter/malic acid transport protein (*Solyc04g080990*; *Solyc09g014610*) and an aluminum-activated malate transporter (*Solyc03g119640*) in drought-stressed tomato (Table 2). As a component of root exudates, malic acid functions in the induction of PGPR chemotaxis and the promotion of biofilm formation for better colonization [21,22]. Therefore, the induction of the malate transporter gene appears to indicate an enhancement in the association between TG1-E1 and the drought-stressed plants.

#### 2.2.4. DEGs Implicating Regulation of Cell Wall Biosynthesis and Organization

Cell wall composition and organization are modified in plants exposed to water deficit [23,24] and bacterial colonization [25]. One of the primary responses of plant cell wall to drought stress is an increase in the biosynthesis of cellulose, xyloglucan and pectin to maintain cell wall integrity and cell turgor pressure, leading to continued cell growth under low water potential [26]. A group of tomato cell wall-related DEGs were identified as regulated by TG1-E1 (Table 3), including two glycosyl hydrolases (*Solyc06g073750*; *Solyc07g006850*) involved in xyloglucan metabolism, a cellulose synthase-like protein (*Solyc03g097050*) and a cellulose synthase-like C1-2 glycosyltransferase (*Solyc02g089640*) implied in cellulose biosynthesis, and a pectin esterase (*Solyc09g075330*) involved in pectin metabolism, thereby implying bacteria-triggered regulation of the cell wall structure in response to drought stress.

In drought-stressed tomato plants, TG1-E1 up-regulated gene expression of several glycosyltransferases (Table 3), which may function in regulating plant cell wall polysaccharides [27]. Particularly, gene up-regulation was observed for a UDP-glucose/GDP-mannose dehydrogenase (*Solyc03g115380*), a GDP-fucose protein O-fucosyltransferase (*Solyc09g011860*), orthologous to *At2g03280*, and a GDP-mannose 4,6 dehydratase (*Solyc12g010540*), which are involved in pectin metabolism [28,29], a potential glucuronoxylan 4-O-methyltransferase-like protein (*Solyc03g110890*), orthologous to *At4g24910*, implied in xylan modifications [30], and a NAD-dependent epimerase/dehydratase (*Solyc06g074670*) that catalyzes the conversion of UDP-D-glucuronate to UDP-D-xylose, providing nucleotide sugars for cell-wall polymers. In addition, a glycosyl hydrolase (*Solyc11g044910*), required for pectic arabinan modification, was down-regulated by TG1-E1. Plant polysaccharides, including arabinogalactan, pectin, and xylan, can act as environmental cues that trigger biofilm formation in certain PGPR strains and consequently support bacterial root colonization [31]. Thus, it is intriguing whether these glycosyltransferases might be involved in TG1-E1 colonization to tomato roots.

We also found a putative matrix metalloproteinase (*Solyc04g005040*) that is potentially involved in the degradation and remodeling of the extracellular matrix, a cell surface continuum beyond the cell wall, which plays a vital role in cell adhesion, cell-cell communication, cell wall modification, and protection against stresses [32]. Altogether, these cell wall-related DEGs indicate that TG1-E1 might trigger modifications in plant cell wall structure and composition, which potentially contributed to TG1-E1 colonization and/or plant adaption to the altered cell turgor pressure.

## 3. Hyperosmotic Stress Induces TG1-E1 Production of Extracellular Metabolites Including Potential Osmoprotectants

To identify the bacterial factors that potentially caused the increased plant drought resistance, we collected TG1-E1 extracellular metabolites under normal and hyperosmotic stressed conditions (PEG30). Subsequently, analysis by gas chromatography coupled with mass spectrometry (GC-MS) identified 49 metabolites as being significantly altered (Fold change ≥ 1.9; *p*-values ≤ 0.05) in TG1-E1 by the hyperosmotic stress (Table 4).

### 3.1. Sugars

TG1-E1 under hyperosmotic stress released higher levels of components of the glycolytic pathway, including glucose-6-P, fructose-6-P, glycerate-3-P, pyruvate and trehalose (Table 4). The glycolytic pathway have been related to increased levels of trehalose as an osmoprotectant molecule against drought in plants [33,34] and bacteria [35,36]. In this sense, we found an increase in trehalose upon PEG treatment, indicating that TG1-E1 might enhance plant drought tolerance by this osmoprotectant sugar.

Under the hyperosmotic stress condition, TG1-E1 also exuded higher levels of xylose, galactose, sucrose, and arabinose (Table 4). These sugars may function as a carbon reservoir, which protects microorganisms from fluctuations in water potential by enhancing water retention and regulating the diffusion of carbon sources [37,38]. Although it is unclear whether the drought-stressed plants would take in these bacteria-derived sugars, these sugars have been identified in other Bacillus strains as bacterial metabolites with a role in improving plant drought resistance [39]. In cells under dehydration stress, the hydroxyl group of cellular sugar alcohols can substitute the hydroxyl group of water to maintain the hydrophilic interactions with the membrane lipids and proteins, thereby helping the maintenance of membrane structural integrity [40].

### 3.2. Amino Acids

TG1-E1 under hyperosmotic stress accumulated a higher level of extracellular arginine, together with several other amino acids (Table 4). Arginine can be used as the substrate for producing polyamines, including spermidine, spermine and their diamine precursor putrescine, which are the major polyamines involved in drought resistance [41,42,43]. Inoculation of *A. thaliana* with *Pseudomonas putida* GAP-P45 caused significant fluctuations in the expression of most polyamine biosynthetic genes and cellular levels of polyamines, including putrescine and spermidine, which positively correlated with the water stress tolerant phenotype of A. thaliana in response to *P. putida* GAP-P45 inoculation [44,45]. In our RNAseq results, TG1-E1 significantly up-regulated the expression of several genes involved in polyamine production, including *Arginine decarboxylase 1* (*SlADC1*; *Solyc10g054440*), which is a rate-limiting enzyme for the biosynthesis of putrescine and other polyamines [46,47], as well as *Adenosylmethionine decarboxylase 2* (*SlSAMDC2*; *Solyc02g089610*) and *S-adenosyl-l-methionine decarboxylase* (*Solyc02g089615*) which are involved in spermidine and spermine biosynthesis [48,49] (Table 5). In contrast, the expression of genes involved in arginine biosynthesis was not affected by TG1-E1. Therefore, it is possible that TG1-E1-produced arginine may be utilized by the plants for polyamine production, thereby contributing to the bacteria-induced plant drought resistance. In addition, TG1-E1 under hyperosmotic stress exuded more spermidine than under the non-stressed condition, indicating that the plants might also directly take up bacterial polyamine to increase drought resistance.

TG1-E1 under hyperosmotic stress accumulated a higher level of extracellular glutamine (Table 4). Meanwhile, TG1-E1 up-regulated gene expression of *Dehydration-responsive element-binding protein 1E-like* (*DREB1a-like*; *Solyc08g007820*) and *Nam-like protein 1* (*Solyc03g080090*) in drought-stressed tomato. These two tomato genes are orthologous to rice *OsDREB1A* and *OsNAC5*, respectively, which are inducible by exogenous glutamine, and play positive roles in rice plant drought resistance [50,51,52]. Thus, it appears that the gene induction of tomato *DREB1a-like* and *Nam-like protein 1* by bacterial glutamine also contributes to TG1-E1-triggered plant drought resistance. Consistent with a positive role of glutamine in plant drought resistance, pepper plants inoculated with *Microbacterium* sp. 3J1 accumulated showed increased levels of glutamine as a major change in metabolites for balancing the hyperosmotic stress [53].

### 3.3. Precursors of the Osmoprotectant Glycine Betaine

The level of ethanolamine in TG1-E1 exudates was increased by hyperosmotic stress (Table 4). Ethanolamine is a precursor of glycine betaine and proline which are two important osmoprotectants in plants [54]. In fact, exogenous application of ethanolamine stimulates glycine betaine biosynthesis in *Nicotiana rustica*, resulting in improved plant resistance to salt stress [55]. TG1-E1 up-regulated gene expression of *Solyc07g008300* (Table 5), which is a choline monooxygenase functioning in the first step of glycine betaine biosynthesis [56]. TG1-E1 up-regulated gene expression of *Solyc08g068600* (Table 5), a putative serine decarboxylase that is involved in ethanolamine metabolism. Meanwhile, the level of TG1-E1 exuded serine was increased by hyperosmotic stress (Table 4). Altogether, these results indicate that TG1-E1-produced ethanolamine may be utilized by the plants for glycine betaine production, thereby contributing to the bacteria-induced plant drought resistance.

### 3.4. Pinitol

An increase in the level of pinitol, a polyol that has been described as an osmoprotectant in several plant species [57,58,59], was also observed in TG1-E1 extracellular metabolites with the hyperosmotic stress treatment (Table 4). Synthesis of D-ononitol, a transient intermediate of D-pinitol, increased in leaves under drought stress conditions in Arabidopsis thaliana; subsequently, the D-ononitol was transported to the roots to synthesize D-pinitol, which then acts as osmoprotectant [58]. Therefore, pinitol produced by TG1-E1 may directly protect tomato roots from drought stress.

Together, these results potentially provide a mechanism for TG1-E1-induced plant drought resistance. It should be noted that in a real system, i.e., plants with root-colonizing bacteria, the dynamic interaction between the roots and the bacteria would likely result in more complex profiles of bacteria extracellular metabolites.

In this study, the plants were transferred from sterile growth medium to a steam-sterilized (20 min at 120 °C) soil mixture of peat and vermiculite (1:1, *v/v*). The plants were then grown in a clean growth chamber. For performing the bacteria inoculation, the bacteria was centrifuged from its original culture solution and then resuspended in sterile 0.45% NaCl solution. Although these actions could not completely prevent unwanted growth of other bacteria, they helped minimize possible involvements of other bacteria. Sometimes the inoculant may fail to colonize and consequently fail to trigger beneficial effects. However, it is unlikely that inoculation of one bacteria strain under these conditions would result in dominant growth of another strain, which then happens to cause the same beneficial effects to the plants. Nonetheless, our study of the soil-grown plants cannot completely exclude the possibility that certain environmental factors other than the inoculant may contribute to the plant-beneficial effects.

## 4. Materials and Methods

### 4.1. Plant Materials and Growth Conditions

Tomato (*Solanum lycopersicum*) seeds of two different cultivars, Micro-Tom and Ailsa Craig, were surface-sterilized by soaking in 5% *v/v* sodium hypochlorite for 5 min. After washing by sterile distilled water, the seeds were planted on 1/2-strength Murashige and Skoog medium (0.5 MS) with 0.7% (*w/v*) agar and 1.5% (*w/v*) sucrose. The plates were then placed in the dark for 4 d at 25 °C. Seedlings were grown under sterile conditions with 200 µmol photons/m2/s light, 25 ± 2 °C, at a 16 h light/8 h dark cycle. Ten days after germination, tomato seedlings were transferred into a steam-sterilized (20 min at 120 °C) soil mixture of peat and vermiculite (1:1, *v/v*). Plant growth and treatments took place in a growth chamber (16 h day at 25 °C + 8 h night at 22 °C).

### 4.2. Bacterial Culture and Inoculation

To make the inoculum, *Bacillus megaterium* TG1-E1 was cultured in liquid Luria–Bertani (LB) medium at 37 °C and 180 rpm overnight. Bacterial growth was estimated by measuring the optical density at 600 nm using a spectrophotometer. The bacterial suspension was centrifuged (5000 rpm, 20 min) and resuspended in 0.45% (*w/v*) NaCl sterile solution. Inoculation with *B. megaterium* TG1-E1 was carried out 2 days after tomato seedlings were planting in 200-mL pots. Each seedling was grown in a separate pot and was inoculated with 50 mL bacterial suspension (10^8^–10^9^ CFU/mL) in 0.45% NaCl sterile solution, and non-inoculated controls were watered with 0.45% NaCl sterile solution.

### 4.3. Monitoring of Plant Growth

After 10 days of bacterial inoculation, the drought treatment was started by stopping the watering. Fresh weight (FW), relative water content (RWC), maximum chlorophyll fluorescence (Fv/Fm) and chlorophyll content of tomato seedlings were measured 10 days after the drought treatment started. The RWC of the leaves was calculated as RWC = (FW − DW) × (TW − DW) − 1. The soil relative humidity was measured by a WET DELTA-T device (DELTA-T DEVICES LTD, Cambridge, England) at 1, 3 and 7 days after the drought treatment started, following the protocol provided by the manufacturer.

### 4.4. Bacteria Root Colonization

Root colonization test was carried out based on Vílchez et al. [60]. Briefly, TG1-E1-inoculated and control tomato seedlings were harvest seven days after disruption of watering. Roots were separated from the aerial portions and were surface-sterilized by immersion in ethanol (75%; vol/vol) for 1 min, then washed for four times using sterile double-distilled water. Separated roots were then grinded using pistils in 1.5 mL tubes. A 0.45% NaCl solution was used to prepare serial dilutions for a drop-by-drop seeding on LB agar plates. After 24 h culturing at 37 °C, bacteria colonies were counted and expressed as c.f.u. mg−1 root dry weight. 

### 4.5. Quantification of Chlorophyll Contents and Photosynthesis Efficiency

Two grams of fresh tomato leaves were immersed in 10 mL of 96% ethanol for 24 h at room temperature in the dark and then centrifuged at 5000 rpm for 10 min at room temperature. The chlorophyll contents of the supernatants were measured by reading optical density (OD) using a Microplate Reader Thermo Varioskan Flash 13. The wave length for optical density measurements were 665 (OD665) and 649 (OD649) nm. The concentrations of total chlorophyll, chlorophyll a, and chlorophyll b were calculated by the following equations:

Chlorophyll a concentration (Ca) = 13.95 × OD665 − 6.88 × OD649.

Chlorophyll b concentration (Cb) = 24.96 × OD649 − 7.32 × OD665.

Total chlorophyll concentration (CT) = Ca + Cb.

Content of chlorophyll = (CT × V)/W, where V is the volume of supernatant and W is the weight of fresh tissue.

The ratio of variable fluorescence/maximum fluorescence (Fv/Fm), which indicates the potential quantum yield of PSII photochemistry, was determined with a Fluorpen FP 100 (Photon Systems Instruments, Brno, Czech Republic) following the protocol provided by Photon Systems Instruments.

### 4.6. Bacterial Extracellular Metabolite Collection and GC-MS Analysis

Extraction of bacterial exudates under regular and hyperosmotic stress (by supplying 30% wt/vol of polyethyleneglycol; PEG-30; 24 h) conditions, were prepared as in [53]. In brief, a 50 mL aliquot of a LB-cultured strain was filtered by a 22 µm mesh, frozen at −80 °C and freeze-dried for further processing. Then, 20 mg per sample were treated with 80% methanol and 1 μM of ribitol was added as an internal control. After being derivatized by using 100 μL methoxylamine hydrochloride in pyridine and treated with 100 μL MSTFA, samples were ready to be run in a TraceGC (up to 1 μL per sample), coupled to a PolarisQ ion trap mass spectrometer (Thermo Finnigan, Dreieich, Germany). Compounds in the sample were separated on a 30 m × 0.25 mm Equity-5 column with 0.25 μm coating of 5% diphenyl 95% dimethylsiloxane (Supelco, Bellefonte, California, CA, USA). Finally, compounds were identified by comparison with purified standards, the NIST 2005 database (NIST, Gaithersburg, MD, USA) and the Golm Metabolome Database. Identities were confirmed by matching mass spectral data and chromatographic retention time. Peak area quantification was prepared with Xcalibur 1.4 software (Thermo Finnigan, Dreieich, Germany) and normalized with ribitol standard and dry mass of the sample. Six replicates per condition were carried out in this analysis.

### 4.7. RNAseq Analysis

Tomato Micro-Tom cultivar was grown and treated as described in the “Plant growth conditions” section. TG1-E1-inoculated and control tomato seedlings were harvested 7 days after disruption of watering. RNA was extracted by using Plant RNeasy Kit (Qiagen, Hilden, Germany), and the integrity was monitored using the RNA Nano 6000 Assay of the Agilent Bioanalyzer 2100 system (Agilent, Santa Clara, CA, USA). RNA purity and concentration were checked using a NanoDrop ND-1000 Spectrophotometer (NanoDrop Technologies, Wilmington, DE, USA).

RNAseq was performed at the Core Facility for Genomics, Shanghai Center for Plant Stress Biology, China. Three biological replicates of each treatment were generated. Total RNA (1 µg) from each sample was used for library preparation with NEBNext Ultra Directional RNA Library Prep Kit for Illumina (New England Biolabs, E7420L), following the manufacturer’s instructions. The prepared libraries were assessed for quality by using NGS High-Sensitivity Kit on a Fragment Analyzer (AATI) and for quantity by using Qubit 2.0 Fluorometer (Thermo Fisher Scientific).

For data analysis, SolexaQA 2.0 [61] and cutadapt v1.10 [62] were used to remove low quality regions and adapter sequences in the raw reads, and clean reads with length longer than 25 bp and phred score greater than 17 were mapped to the Tair10 reference genome using TopHat 2.0.10 [63] with default parameters. The reads that were mapped to each annotated gene were counted by HTseq-count (version 0.9.1) [64]. Using edgeR [65], the raw counts of each gene were normalized, and differentially expressed genes were identified using fold change >2 and false discovery rate < 0.01 as significance cutoffs. Three biological replicates of each condition were used to perform transcriptome analyses.

### 4.8. Statistical Analysis

Statistical analyses were performed with Prism software (https://www.graphpad.com/scientific-software/prism/, Accessed 4 April 2020). Significant difference between treatments was based on *p*-values ≤ 0.05.

## 5. Conclusions

PGPR are promising tools for increasing crop drought resistance. In this study, we show that *B. megaterium* TG1-E1 increases drought resistance in different tomato cultivars. Our transcriptome analysis reveals several key mediators of TG1-E1-induced transcriptional regulation in tomato plants, including transcription factors, stress signaling components and regulators, and putative regulators of cell wall organization. In addition, our analysis of TG1-E1 extracellular metabolites identifies some important compounds, including sugars, amino acid, ethanolamine, and pinitol, which are potentially regulators (or precursors of regulators) of TG1-E1-triggered plant drought resistance. These findings not only contribute to our understanding of PGPR-triggered drought resistance in tomato plants, but also provide important clues for future elucidation of the underlying molecular mechanisms.

## Figures and Tables

**Table 1:** Transcription factors that were transcriptionally regulated by TG1-E1 in drought-stressed tomato plants (Micro-Tom). The differentially expressed genes (DEGs) were defined by log2 fold-change values (LogFC) > 1 or < −1 with FDR < 0.01.

Gene ID	Annotation	Type of Nucleic Acid Binding Transcription Factor Activity	LogFC
*Solyc07g061760.2*	Ankyrin repeats	Ankyrin repeat-containing domain	2.29
*Solyc08g014570.3*	PGG domain	Ankyrin repeat-containing domain	1.06
*Solyc06g076050.3*	Ankyrin repeats	Ankyrin repeat-containing domain	1.04
*Solyc05g052570.3*	Ankyrin repeats	Ankyrin repeat-containing domain	1.11
*Solyc05g013540.1*	AP2 domain	AP2/ERF domain	1.07
*Solyc02g090770.1*	AP2 domain	AP2/ERF domain	2.79
*Solyc08g080290.3*	AP2 domain	AP2/ERF domain	2.58
*Solyc08g007830.1*	AP2 domain	AP2/ERF domain	4.48
*Solyc06g035700.1*	AP2 domain	AP2/ERF domain	3.41
*Solyc08g007820.1*	AP2 domain	AP2/ERF domain	3.02
*Solyc10g050970.1*	AP2 domain	AP2/ERF domain	3.01
*Solyc01g108240.3*	AP2 domain	AP2/ERF domain	4.22
*Solyc10g086530.1*	GRAS domain family	GRAS domain	1.10
*Solyc11g012510.2*	GRAS domain family	GRAS domain	1.28
*Solyc12g005340.2*	GRAS domain family	GRAS domain	1.77
*Solyc08g080540.3*	HSF-type DNA-binding	HSF-type DNA binding	1.64
*Solyc02g089200.3*	SRF-type transcription factor	MADS-box	1.95
*Solyc05g015750.3*	SRF-type transcription factor	MADS-box	1.67
*Solyc01g093960.3*	SRF-type transcription factor	MADS-box	1.04
*Solyc06g059970.3*	SRF-type transcription factor	MADS-box	1.03
*Solyc02g071730.3*	SRF-type transcription factor	MADS-box	1.09
*Solyc08g067230.3*	K-box region	MADS-box/K-box	2.25
*Solyc04g081000.3*	K-box region	MADS-box/K-box	1.42
*Solyc05g015840.3*	SBP domain	SBP-box	1.17
*Solyc10g011910.3*	WRKY DNA -binding domain	Zinc finger domain	1.84
*Solyc03g121400.1*	Dof domain, zinc finger	Zinc finger domain	1.10
*Solyc06g053640.1*	Ring finger domain	Zinc finger domain	1.85
*Solyc10g009550.3*	WRKY DNA -binding domain	Zinc finger domain	1.40
*Solyc08g008280.3*	WRKY DNA -binding domain	Zinc finger domain	1.28
*Solyc04g015360.3*	GATA zinc finger	Zinc finger domain	1.04
*Solyc06g075780.2*	C2H2-type zinc finger	Zinc finger domain	1.49
*Solyc05g051200.1*	AP2 domain	AP2/ERF domain	−1.41
*Solyc04g007000.1*	AP2 domain	AP2/ERF domain	−1.80
*Solyc03g119390.3*	Helix-loop-helix DNA-binding domain	bHLH domain	−1.55
*Solyc03g034000.3*	Helix-loop-helix DNA-binding domain	bHLH domain	−1.53
*Solyc12g049320.2*	GRAS domain family	GRAS domain	−1.34
*Solyc02g077590.1*	Homeobox domain	HD-Zip domain	−1.34
*Solyc05g048830.3*	Myb-like DNA-binding domain	Myb domain	−1.05
*Solyc05g053330.3*	Myb-like DNA-binding domain	Myb domain	−1.12
*Solyc12g089190.1*	Myb-like DNA-binding domain	Myb domain	−1.27
*Solyc12g008800.2*	Myb-like DNA-binding domain	Myb domain	−1.42
*Solyc04g076220.3*	Domain of unknown function	PPC domain	−1.30
*Solyc05g006340.3*	WD domain, G-beta repeat	WD domain	−1.16
*Solyc11g006450.2*	Ring finger domain	Zinc finger domain	−1.22
*Solyc05g054650.1*	C2H2-type zinc finger	Zinc finger domain	−1.24
*Solyc05g051860.3*	zinc-finger of the FCS-type, C2-C2	Zinc finger domain	−1.33
*Solyc05g015850.3*	WRKY DNA -binding domain	Zinc finger domain	−1.48
*Solyc06g008020.3*	HIT zinc finger	Zinc finger domain	−1.15
*Solyc00g136260.1*	Ring finger domain	Zinc finger domain	−1.97
*Solyc12g008830.2*	GATA zinc finger	Zinc finger domain	−1.44
*Solyc03g032060.1*	Ring finger domain	Zinc finger domain	−1.11
*Solyc12g006230.2*	Ring finger domain	Zinc finger domain	−1.77
*Solyc08g067360.3*	WRKY DNA -binding domain	Zinc finger domain	−1.39
*Solyc07g045180.3*	B-box zinc finger	Zinc finger domain	−1.23
*Solyc08g082680.3*	Ring finger domain	Zinc finger domain	−1.00
*Solyc08g067970.3*	zinc-finger of the FCS-type, C2-C2	Zinc finger domain	−1.11
*Solyc06g061240.3*	PLATZ transcription factor	Zinc finger domain	−1.08
*Solyc08g065940.3*	Zinc finger C-x8-C-x5-C-x3-H type	Zinc finger domain	−1.08

**Table 2:** Signaling-related proteins that were transcriptionally regulated by TG1-E1 in drought-stressed tomato plants (Micro-Tom). The differentially expressed genes (DEGs) were defined by log2 fold-change values (LogFC) > 1 or < −1 with FDR < 0.01.

Gene ID	Annotation	Type of Signaling Protein	LogFC
*Solyc11g072930.2*	Carbohydrate-binding protein of the ER	RPK	1.74
*Solyc06g062450.3*	Carbohydrate-binding protein of the ER	RPK	1.44
*Solyc02g094010.2*	Protein kinase domain	RPK	1.58
*Solyc09g014590.3*	Leucine Rich Repeat	RPK	3.12
*Solyc10g006690.3*	Protein tyrosine kinase	RPK	1.01
*Solyc06g007190.3*	Protein phosphatase 2C	ILKAP	2.04
*Solyc12g009550.1*	Leucine rich repeat	RPK	1.07
*Solyc01g005730.3*	Leucine rich repeat	RPK	1.14
*Solyc08g077630.3*	Protein kinase domain	RPK	1.54
*Solyc01g098690.2*	Leucine rich repeat N-terminal domain	RPK	1.23
*Solyc05g053010.1*	Protein kinase domain	RPK	1.59
*Solyc07g064820.1*	Protein kinase domain	MAPK	1.02
*Solyc11g020230.1*	Protein kinase domain	RPK	1.05
*Solyc02g064980.1*	Protein kinase domain	MAPK	3.15
*Solyc02g090970.1*	Protein kinase domain	MAPK	3.05
*Solyc08g077560.3*	Protein tyrosine kinase	RPK	1.42
*Solyc04g074270.3*	Leucine rich repeat	RPK	1.08
*Solyc02g089900.1*	LysM domain	RPK	1.07
*Solyc06g005170.3*	Protein kinase domain	MAPK	1.65
*Solyc09g018280.1*	NAF domain	CDPK	1.14
*Solyc02g092450.3*	E1-E2 ATPase	PM-CA-ATPase	1.28
*Solyc02g064680.3*	E1-E2 ATPase	PM-CA-ATPase	1.87
*Solyc01g099370.3*	C2 domain	CaLB	1.61
*Solyc08g008370.3*	Development and cell death domain	CaLB	1.66
*Solyc03g113980.3*	Calmodulin binding protein-like	CLM	1.05
*Solyc06g053930.3*	EF-hand domain pair	CaM	1.04
*Solyc03g097100.1*	EF-hand domain pair	CLM	1.10
*Solyc11g071740.2*	EF-hand domain pair	CLM	2.67
*Solyc03g118810.1*	EF-hand domain pair	CaM	1.38
*Solyc03g119250.3*	Calmodulin binding protein-like	CLM	1.99
*Solyc06g006020.2*	Leucine Rich Repeat	RPK	−1.31
*Solyc12g009510.1*	Leucine rich repeat	RPK	−1.13
*Solyc11g017280.2*	Leucine rich repeat	RPK	−1.07
*Solyc04g074020.2*	Leucine rich repeat N-terminal domain	RPK	−1.75
*Solyc04g009910.3*	Protein kinase domain	CDPK	−1.21
*Solyc12g009780.1*	Leucine Rich Repeat	RPK	−1.19
*Solyc04g074030.3*	Leucine rich repeat N-terminal domain	RPK	−1.91
*Solyc11g011180.2*	Leucine Rich repeat	RPK	−1.23
*Solyc05g012430.1*	Leucine rich repeat	RPK	−1.71
*Solyc09g090210.3*	Protein tyrosine kinase	RPK	−1.10
*Solyc07g006770.2*	TMEM154 protein family	RPK	−1.20
*Solyc10g076550.1*	Protein kinase domain	RPK	−1.85
*Solyc04g074050.3*	Protein kinase domain	RPK	−1.75
*Solyc01g067020.3*	Protein kinase domain	RPK	−1.16
*Solyc09g075920.1*	D-mannose binding lectin	RPK	−1.10
*Solyc08g016270.2*	Leucine rich repeat	RPK	−1.13
*Solyc04g074000.3*	Protein tyrosine kinase	RPK	−1.64
*Solyc08g077730.3*	MORN repeat	PIPK	−1.22
*Solyc04g009900.3*	Protein kinase domain	PPCK	−1.27
*Solyc12g088840.1*	EF-hand domain	CaM	−1.03
*Malic acid related DEGs*			
*Solyc04g080990.2*	Voltage-dependent anion channel		1.32
*Solyc09g014610.3*	Voltage-dependent anion channel		1.04
*Solyc03g119640.3*	Aluminum activated malate transporter		1.30

RPK: Receptor-like kinase protein. ILKAP: Integrin-linked kinase-associated serine/threonine protein. MAPK: Mitogen-activated protein kinase. CDPK: Calcium/calmodulin-dependent protein kinase protein. PM-CA-ATPase: Plasma membrane Ca^2+^ ATPase protein. CaLB: Calcium-dependent lipid-binding protein. CaM: Calmodulim protein. CLM: Calmodulin-binding protein-like. PIPK: Phosphoinositide kinase-like protein. PPCK: Phosphoenolpyruvate carboxylase kinase.

**Table 3:** Cell wall-related proteins that were transcriptionally regulated by TG1-E1 in drought-stressed tomato plants (Micro-Tom). The differentially expressed genes (DEGs) were defined by log2 fold-change values (LogFC) > 1 or < −1 with FDR < 0.01. UDP: Uridine diphosphate; GDP: Guanosine diphosphate; NAD: Nicotinamide adenine dinucleotide.

Gene ID	Annotation	LogFC
*Solyc06g073750.3*	Glycosyl hydrolase	1.99
*Solyc07g006850.2*	Glycosyl hydrolase	1.04
*Solyc03g115380.2*	UDP-glucose/GDP-mannose dehydrogenase	1.78
*Solyc02g094010.2*	Protein kinase domain	1.58
*Solyc03g110890.1*	Polysaccharide biosynthesis related protein	1.13
*Solyc09g075330.3*	Pectinesterase	1.08
*Solyc09g011860.3*	GDP-fucose protein O-fucosyltransferase	1.69
*Solyc12g010540.1*	GDP-mannose 4,6 dehydratase	1.07
*Solyc04g005040.1*	Putative peptidoglycan binding domain	1.37
*Solyc02g089640.3*	Glycosyltransferase-like	1.03
*Solyc03g123620.4*	Plant invertase/pectin methylesterase inhibitor	1.35
*Solyc03g097050.3*	RING/Ubox-like zinc-binding domain	1.42
*Solyc06g074670.3*	NAD dependent epimerase/dehydratase	1.31
*Solyc04g072280.3*	Multicopper oxidase	−2.95
*Solyc03g026360.1*	LysM domain	−1.23
*Solyc11g044910.2*	Glycosyl hydrolase domain	−1.66

**Table 4:** Extracellular metabolites released from *B. megaterium* TG1-E1. The ratios show metabolite levels from TG1-E1 with hyperosmotic stress (by PEG30) compared to the non-stressed condition. N = 3 biological replicates. All ratios have *p* < 0.05, Student’s *t*-test. MeOX: methoximated-derived compound.

Compound	Mock	PEG30	LogFC
Piruvate	0.04	0.14	1.72
beta-Aminoisobutiric acid	3.99	11.99	1.59
acid α-ketocaproate	0.35	2.88	3.06
Urea	20.53	55.66	1.44
Ethanolamine	2.11	15.58	2.88
Leucine	2666.87	6457.33	1.28
Succinate	51.39	130.40	1.34
Glycerate	6.04	16.87	1.48
Fumarate	4.64	9.08	0.97
Serine	7.56	34.32	2.18
Threonine	8.33	50.54	2.60
beta-Alanine	1.19	17.05	3.84
Glutamine	1.51	6.59	2.13
Malate	2.20	5.55	1.33
Cytosine	0.05	0.17	1.72
L-Aspartate	48.05	160.37	1.74
Ornithin-Citrullin-Arginine	1.09	4.55	2.06
Xylose MeOX1	0.12	1.55	3.65
Ribose	0.22	0.64	1.54
cis-Aconitate	0.18	0.56	1.65
1-Methyl-L-histidine	0.11	0.49	2.19
Glycerate-3-P	0.40	0.97	1.28
Citrullin-Ornithin-Arginine	6.14	78.11	3.67
Citrate	2.13	6.83	1.68
Isocitrate	0.52	1.45	1.47
Arginine-NH_3_	8.62	16.92	0.97
Pinitol	0.10	0.28	1.50
Fructose MEOX1	0.09	0.30	1.78
Fructose MEOX2	0.04	0.12	1.52
Galactose MeOX1	0.11	0.38	1.84
Glucose MEOX1	0.46	1.46	1.67
Glucose MEOX2	0.04	0.17	1.99
Glucuronic acid MEOX1	0.03	0.13	2.04
Gluconate	0.23	0.59	1.35
myo-Inositol	7.21	24.71	1.78
Spermidine	0.45	3.41	2.93
L-Cystathionine	0.71	3.80	2.42
Tryptophan	112.05	222.43	0.99
Fructose-6-P	0.04	0.09	1.18
Gluconate-6-P	0.03	0.13	2.17
Sucrose	0.59	2.00	1.75
Trehalose	0.33	0.66	0.98
Lysine	4737.93	12276.77	1.37
Histidine	193.44	393.00	1.02
Maleic acid	9.10	2.61	−1.80
Uracil	51.66	13.95	−1.89
Thymine	6.94	0.16	−5.46
4-Aminobutyrate (GABA)	20.49	3.28	−2.64

**Table 5:** Transcriptional regulation of gene potentially regulated by TG1-E1 extracellular metabolites under hyperosmotic stress. The differentially expressed genes (DEGs) were defined by log2 fold-change values (LogFC) > 1 or < −1 with FDR < 0.01.

Gene ID	Annotation	LogFC
*Solyc10g054440.2*	Pyridoxal-dependent decarboxylase	1.46
*Solyc02g089610.2*	Adenosylmethionine decarboxylase	1.39
*Solyc02g089615.1*	S-adenosyl-l-methionine decarboxylase leader peptide	1.31
*Solyc08g007820.1*	Dehydration-responsive element-binding protein 1E-like	3.02
*Solyc03g080090.3*	No apical meristem (NAM) protein	1.08
*Solyc07g008300.2*	Ring hydroxylating alpha subunit	2.28
*Solyc08g068600.3*	Pyridoxal-dependent decarboxylase conserved domain	1.20

## Data Availability

Data is contained within the article or Supplementary Material.

## Supplementary Materials

The following are available online at https://www.mdpi.com/article/10.3390/metabo11060369/s1, Figure S1: Bacillus megaterium TG1-E1 alleviate drought stress and maintain growth in Ailsa-Craig tomato plants; Figure S2: Root colonization by TG1-E1 in tomato seedlings exposed to drought. Figure S3: Distribution of enriched Gene Ontology (GO). Supplementary Table S1: Differentially expressed genes (DEGs) regulated by TG1-E1 in drought stressed tomato plants (Micro-Tom) compared to un-inoculated plants. Excel files: Supplementary table Metabolites and Supplementary table RNAseq.
