# Supplementary material for: Plant Transcriptome Reprograming and Bacterial Extracellular Metabolites Underlying Tomato Drought Resistance Triggered by a Beneficial Soil Bacteria
Source: Metabolites. 2021 Jun 9;11(6):369. doi: 10.3390/metabo11060369 (PMC8230097; doi:10.3390/metabo11060369)
Supplement: Supplementary file 1 [file metabolites-11-00369-s001.zip › Supplementary Material.pdf]

Figure S1. ***Bacillus megaterium*** **TG1-E1 alleviate drought stress and maintain growth in Ailsa-Craig tomato plants.** **(A)** TG1-E1 increased drought resistance in tomato (cultivar Ailsa-Craig) plants. Images were taken at 10 days after the drought treatment (DAT). **(B)** Measurements of plant fresh weight. **(C)** Quantification of photosynthesis efficiency (Fv/Fm). **(D)** Measurements of chlorophyll contents. **(E)** Relative humidity of the soil where tomato seedlings were grown. Results in panels B, C, and D were from plants harvested at 10 DAT. The bar graphs show representative results from three independent experiments. Mean ± SE (n = 6 biological replicates). Asterisk denote significant differences at p <0.05, Tukey's multiple comparison test.

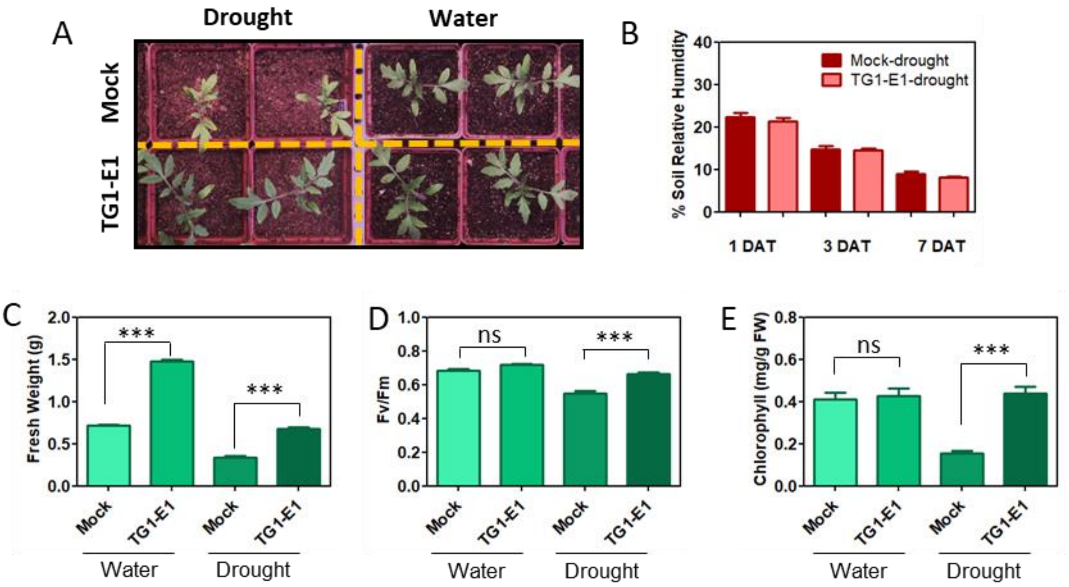

Supplementary Figure S2. Root colonization by TG1-E1 in tomato seedlings exposed to drought. Root colonization of *B. megaterium* TG1-E1 in Micro-Tom tomato seedlings grown in well watered conditions (Water) and seven days after disruption of water (Drought). Different letters denote significantly different means at P < 0.05, Tukey's multiple comparison test.

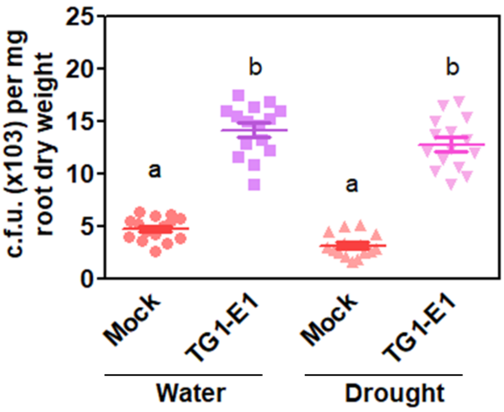

Supplementary Figure S3. Distribution of enriched Gene Ontology (GO). Significant enriched GO terms for Biological Process (green) and Molecular Function (blue) of TG1-E1-inocualted tomato seedlings subjected to drought compared to un-inoculated plants for up- and down-regulated differentially expressed genes (DEGs). Significance scores were calculated as the -log10 of the p-value. Enriched GO categories were determined by using AgriGOv2 software.

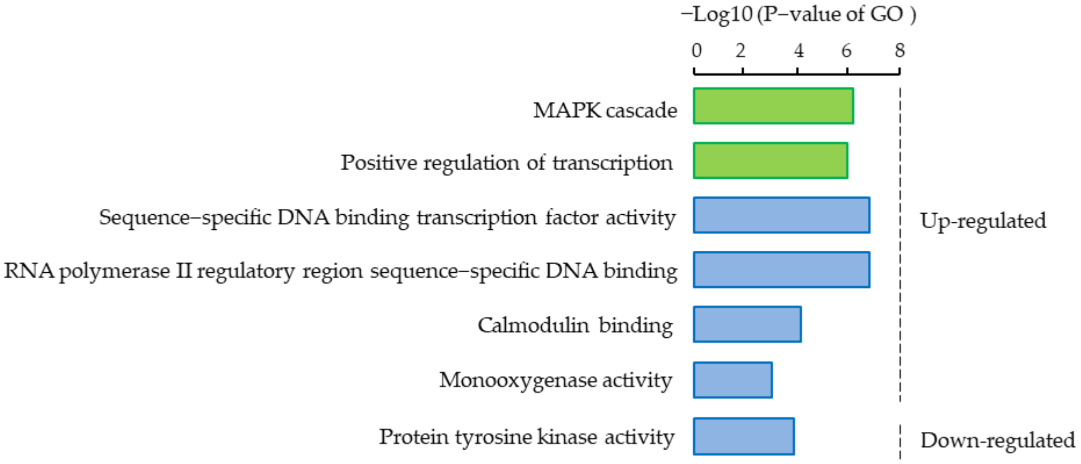

Supplementary Table S1. Differentially expressed genes (DEGs) regulated by TG1-E1 in drought stressed tomato plants (Micro-Tom) compared to un-inoculated plants. The differentially expressed genes (DEGs) were defined by log2 fold-change values (LogFC) > 1 or < -1 with FDR < 0.01.

Gene ID	LogFC	Annotation
Up-regulated		
Solyc03g058370.2	3.67	UDP-glucoronosyl and UDP-glucosyl transferase
Solyc06g009890.2	3.60	no_annotation
Solyc06g073750.3	1.99	Glycosyl hydrolase family 3 N terminal domain
Solyc07g061750.3	3.21	Domain of unknown function
Solyc08g062580.1	3.63	PPR repeat
Solyc08g068410.2	4.81	no_annotation
Solyc09g005500.3	3.60	Pathogenesis-related protein Bet v I family
Solyc10g007860.3	3.04	Cytochrome P450
Solyc11g013410.2	3.51	SNF5 / SMARCB1 / INI1
Solyc11g013430.2	5.16	SNF5 / SMARCB1 / INI1
Solyc12g077660.2	2.50	no_annotation
Solyc08g023340.3	2.38	no_annotation
Solyc10g046920.1	2.54	no_annotation
Solyc01g096750.1	2.07	Argonaute linker 2 domain
Solyc07g061760.2	2.30	Ankyrin repeats (3 copies)
Solyc12g035420.2	3.29	no_annotation
Solyc01g091870.3	1.03	Major Facilitator Superfamily
Solyc05g015350.3	1.10	Cytochrome P450
Solyc09g082340.2	2.18	Cupin

Solyc07g009280.3	1.13	no_annotation
Solyc07g042400.2	1.26	no_annotation
Solyc08g067230.3	2.25	K-box region
Solyc02g089200.3	1.95	SRF-type transcription factor (DNA-binding and dimerisation domain)
Solyc05g015750.3	1.67	SRF-type transcription factor (DNA-binding and dimerisation domain)
Solyc04g080990.2	1.32	Voltage-dependent anion channel
Solyc11g005240.1	2.06	SRR1
Solyc12g098930.2	1.44	Mitochondrial branched-chain alpha-ketoacid dehydrogenase kinase
Solyc06g068350.1	1.71	no_annotation
Solyc08g079970.1	1.14	PA domain
Solyc03g113980.3	1.06	Calmodulin binding protein-like
Solyc06g005620.3	1.17	ZIP Zinc transporter
Solyc06g072012.1	1.62	no_annotation
Solyc07g006850.2	1.04	Glycosyl hydrolases family 16
Solyc07g061930.1	2.52	no_annotation
Solyc09g056040.3	2.13	HECT-domain (ubiquitin-transferase)
Solyc11g072930.2	1.74	Carbohydrate-binding protein of the ER
Solyc06g062450.3	1.44	Carbohydrate-binding protein of the ER
Solyc06g071337.1	1.26	no_annotation
Solyc06g076870.3	2.18	60s Acidic ribosomal protein
Solyc07g008300.2	2.28	Ring hydroxylating alpha subunit (catalytic domain)
Solyc02g043940.1	1.28	no_annotation
Solyc12g017264.1	3.01	no_annotation
Solyc00g026160.3	1.36	Ferric reductase NAD binding domain
Solyc02g093070.3	1.02	non-haem dioxygenase in morphine synthesis N-terminal
Solyc03g116795.1	3.19	no_annotation
Solyc07g043060.1	1.73	UDP-glucoronosyl and UDP-glucosyl transferase
Solyc04g014770.1	1.23	no_annotation
Solyc12g100110.1	1.26	no_annotation
Solyc12g040320.2	3.27	no_annotation
Solyc03g115380.2	1.78	UDP-glucose/GDP-mannose dehydrogenase family, NAD binding domain
Solyc04g081000.3	1.43	K-box region
Solyc03g025250.3	2.01	MatE
Solyc07g008107.1	2.19	Plastocyanin-like domain
Solyc01g093960.3	1.04	SRF-type transcription factor (DNA-binding and dimerisation domain)
Solyc02g094010.2	1.58	Protein kinase domain
Solyc04g055150.1	1.02	no_annotation
Solyc05g013540.1	1.07	AP2 domain
Solyc08g075190.1	1.02	no_annotation
Solyc02g031950.3	1.71	no_annotation
Solyc05g032620.1	1.44	no_annotation
Solyc03g097530.3	1.05	no_annotation
Solyc04g056380.3	1.20	Putative adipose-regulatory protein (Seipin)
Solyc08g048410.2	1.26	Ubiquitin carboxyl-terminal hydrolase

Solyc02g094110.1	1.53	Cytochrome P450
Solyc01g107810.2	1.27	UDP-glucoronosyl and UDP-glucosyl transferase
Solyc02g090770.1	2.79	AP2 domain
Solyc01g103630.3	2.43	no_annotation
Solyc03g098790.3	1.20	Trypsin and protease inhibitor
Solyc08g007805.1	2.75	CHCH domain
Solyc02g091990.3	2.02	Aminotransferase class I and II
Solyc06g053930.3	1.04	EF-hand domain pair
Solyc12g006360.2	1.72	MatE
Solyc12g042960.2	1.17	Protein of unknown function (DUF2921)
Solyc02g066970.1	1.07	no_annotation
Solyc07g009040.3	2.50	no_annotation
Solyc03g097100.1	1.11	EF-hand domain pair
Solyc06g059970.3	1.03	SRF-type transcription factor (DNA-binding and dimerisation domain)
Solyc02g071730.3	1.09	SRF-type transcription factor (DNA-binding and dimerisation domain)
Solyc02g090500.3	1.42	no_annotation
Solyc04g025750.3	1.14	EamA-like transporter family
Solyc10g051080.1	1.57	no_annotation
Solyc02g089860.1	1.36	GNS1/SUR4 family
Solyc09g014590.3	3.12	Leucine Rich Repeat
Solyc08g080540.3	1.64	HSF-type DNA-binding
Solyc10g045433.1	1.11	no_annotation
Solyc01g102380.4	1.31	Cupin
Solyc07g009030.3	1.77	no_annotation
Solyc07g065165.1	1.09	Pirin C-terminal cupin domain
Solyc01g098790.2	1.25	no_annotation
Solyc02g084980.3	1.03	Glycosyl transferase family 8
Solyc03g110890.1	1.13	Polysaccharide biosynthesis
Solyc03g116335.1	1.29	no_annotation
Solyc09g005940.3	1.44	Male sterility protein
Solyc10g006690.3	1.00	Protein tyrosine kinase
Solyc03g115780.2	1.33	Exonuclease
Solyc09g005570.3	1.53	DUF761-associated sequence motif
Solyc12g044190.2	3.32	NB-ARC domain
Solyc10g050670.1	1.34	no_annotation
Solyc06g007190.3	2.04	Protein phosphatase 2C
Solyc09g075330.3	1.08	Pectinesterase
Solyc08g080290.3	2.58	AP2 domain
Solyc09g018610.1	1.21	no_annotation
Solyc08g036625.1	4.77	no_annotation
Solyc10g085870.1	1.09	UDP-glucoronosyl and UDP-glucosyl transferase
Solyc10g086530.1	1.10	GRAS domain family
Solyc12g009550.1	1.07	Leucine rich repeat
Solyc03g111280.1	4.30	Cytochrome P450
Solyc03g116820.3	1.45	no_annotation
Solyc02g014840.3	1.50	no_annotation

Solyc10g086690.2	2.35	PAP2 superfamily C-terminal
Solyc08g081555.1	1.08	Aminotransferase class I and II
Solyc08g036620.3	4.24	Divergent CCT motif
Solyc03g117260.2	2.95	Plant protein of unknown function
Solyc06g066520.1	1.28	Cotton fibre expressed protein
Solyc01g005730.3	1.15	Leucine rich repeat
Solyc06g075130.3	1.58	START domain
Solyc10g084000.2	1.99	Heavy-metal-associated domain
Solyc08g007830.1	4.48	AP2 domain
Solyc12g044700.2	3.27	no_annotation
Solyc08g077630.3	1.54	Protein kinase domain
Solyc10g084880.3	1.24	Domain of unknown function (DUF3475)
Solyc12g042700.2	3.50	no_annotation
Solyc10g011910.3	1.84	WRKY DNA -binding domain
Solyc08g036660.3	4.48	tify domain
Solyc07g041620.1	1.09	no_annotation
Solyc02g088740.1	1.11	no_annotation
Solyc05g013410.3	1.59	F-box-like
Solyc08g062680.1	1.36	DDE superfamily endonuclease
Solyc12g010420.1	1.56	no_annotation
Solyc06g035700.1	3.41	AP2 domain
Solyc03g116830.3	1.30	PX domain
Solyc11g006540.2	1.04	Pyridine nucleotide-disulphide oxidoreductase
Solyc12g087790.1	1.10	no_annotation
Solyc03g114710.3	1.01	UDP-glucoronosyl and UDP-glucosyl transferase
Solyc08g007820.1	3.02	AP2 domain
Solyc03g121400.1	1.10	Dof domain, zinc finger
Solyc03g111300.1	4.13	Cytochrome P450
Solyc10g050970.1	3.01	AP2 domain
Solyc04g072920.3	1.49	Trehalose-phosphatase
Solyc01g098800.3	1.11	Sodium/calcium exchanger protein
Solyc01g106290.3	1.24	no_annotation
Solyc02g087950.3	1.25	Protein of unknown function (DUF1666)
Solyc06g053640.1	1.85	Ring finger domain
Solyc02g089615.1	1.31	S-adenosyl-l-methionine decarboxylase leader peptide
Solyc09g014610.3	1.04	Voltage-dependent anion channel
Solyc01g098690.2	1.23	Leucine rich repeat N-terminal domain
Solyc03g094160.3	1.01	Caspase domain
Solyc10g009550.3	1.40	WRKY DNA -binding domain
Solyc11g071740.2	2.67	EF-hand domain pair
Solyc05g015840.3	1.17	SBP domain
Solyc08g008280.3	1.29	WRKY DNA -binding domain
Solyc05g053010.1	1.60	Protein kinase domain
Solyc07g064820.1	1.02	Protein kinase domain
Solyc01g079200.3	2.87	non-haem dioxygenase in morphine synthesis N-terminal
Solyc07g008710.3	2.81	Pathogenesis-related protein Bet v I family
Solyc03g036470.2	1.99	Aromatic amino acid lyase
Solyc03g119640.3	1.30	Aluminium activated malate transporter

Solyc12g010440.2	1.19	Major Facilitator Superfamily
Solyc02g068680.1	1.08	C1 domain
Solyc08g068350.3	1.01	no_annotation
Solyc11g020230.1	1.05	Protein kinase domain
Solyc07g007170.3	1.31	no_annotation
Solyc02g062550.3	1.69	ATPase family associated with various cellular activities (AAA)
Solyc04g015360.3	1.04	GATA zinc finger
Solyc02g064980.1	3.15	Protein kinase domain
Solyc03g117270.1	1.60	F-box domain
Solyc02g090970.1	3.05	Protein kinase domain
Solyc08g036640.3	4.28	Divergent CCT motif
Solyc02g092450.3	1.28	E1-E2 ATPase
Solyc08g068600.3	1.20	Pyridoxal-dependent decarboxylase conserved domain
Solyc02g078890.1	2.12	no_annotation
Solyc02g094400.3	1.14	Glycerophosphoryl diester phosphodiesterase family
Solyc01g106390.3	1.03	Shikimate / quinate 5-dehydrogenase
Solyc04g081530.1	1.61	DnaJ domain
Solyc08g014570.3	1.07	Domain of unknown function
Solyc10g005480.3	1.13	no_annotation
Solyc02g084890.2	1.12	NB-ARC domain
Solyc10g085590.1	1.15	Vps23 core domain
Solyc11g012510.2	1.28	GRAS domain family
Solyc09g018280.1	1.14	NAF domain
Solyc05g052560.1	1.24	no_annotation
Solyc02g070040.1	2.24	no_annotation
Solyc03g116340.3	1.07	no_annotation
Solyc01g107290.3	1.62	Ring finger domain
Solyc02g090380.3	1.17	Arabidopsis broad-spectrum mildew resistance protein RPW8
Solyc01g102840.3	1.76	TIR domain
Solyc08g077020.1	1.61	Auxin responsive protein
Solyc10g049630.2	1.21	Protein phosphatase 2C
Solyc06g075780.2	1.49	C2H2-type zinc finger
Solyc02g087480.3	1.12	Protein of unknown function (DUF1336)
Solyc08g077560.3	1.42	Protein tyrosine kinase
Solyc01g108240.3	4.22	AP2 domain
Solyc02g071220.3	1.04	Response regulator receiver domain
Solyc08g062690.2	1.38	no_annotation
Solyc12g006420.2	1.22	no_annotation
Solyc06g076020.3	1.77	Hsp70 protein
Solyc01g111440.3	1.28	Senescence-associated protein
Solyc03g118470.3	1.04	NUDIX domain
Solyc09g011860.3	1.69	GDP-fucose protein O-fucosyltransferase
Solyc06g076040.3	1.21	U-box domain
Solyc12g010540.1	1.07	GDP-mannose 4,6 dehydratase
Solyc04g074430.1	1.11	Phosphate-induced protein 1 conserved region
Solyc02g091500.1	1.40	EF-hand domain pair

Solyc04g005040.1	1.37	Putative peptidoglycan binding domain
Solyc04g005480.1	1.24	no_annotation
Solyc10g081570.2	1.76	no_annotation
Solyc02g089610.2	1.40	Adenosylmethionine decarboxylase
Solyc12g009000.1	1.86	DDE superfamily endonuclease
Solyc05g052550.1	1.26	no_annotation
Solyc07g032230.3	1.10	Regulator of Vps4 activity in the MVB pathway
Solyc06g076050.3	1.04	Ankyrin repeat
Solyc07g006890.1	1.91	Cytochrome P450
Solyc01g087590.3	2.56	Flavin containing amine oxidoreductase
Solyc01g094410.3	1.06	Plant phosphoribosyltransferase C-terminal
Solyc10g086280.2	1.51	Heavy-metal-associated domain
Solyc01g095140.3	2.52	Late embryogenesis abundant protein
Solyc04g074450.1	1.08	Phosphate-induced protein 1 conserved region
Solyc02g077470.3	1.02	Optic atrophy 3 protein (OPA3)
Solyc03g120550.2	1.06	POT family
Solyc02g087540.2	1.69	Domain associated at C-terminal with AAA
Solyc04g074270.3	1.08	Leucine rich repeat
Solyc04g079730.1	2.69	Cytochrome P450
Solyc02g089900.1	1.07	LysM domain
Solyc02g089640.3	1.03	Glycosyltransferase like family 2
Solyc11g018777.1	1.85	Peroxidase
Solyc06g049020.1	1.64	no_annotation
Solyc12g005340.2	1.77	GRAS domain family
Solyc06g009140.3	1.08	Late embryogenesis abundant protein
Solyc06g073050.2	1.60	No apical meristem (NAM) protein
Solyc06g075690.3	2.16	Domain of unknown function (DUF966)
Solyc01g099370.3	1.61	C2 domain
Solyc03g123620.4	1.35	Plant invertase/pectin methylesterase inhibitor
Solyc12g009220.2	2.57	tify domain
Solyc07g040960.1	1.59	DDE superfamily endonuclease
Solyc08g008370.3	1.66	Development and cell death domain
Solyc05g052570.3	1.12	Ankyrin repeats (3 copies)
Solyc03g080090.3	1.08	No apical meristem (NAM) protein
Solyc03g118810.1	1.38	EF-hand domain pair
Solyc03g119250.3	2.00	Calmodulin binding protein-like
Solyc03g097050.3	1.42	RING/Ubox like zinc-binding domain
Solyc06g005170.3	1.65	Protein kinase domain
Solyc03g122190.3	2.33	Divergent CCT motif
Solyc01g095150.3	1.57	Late embryogenesis abundant protein
Solyc06g074670.3	1.31	NAD dependent epimerase/dehydratase family
Solyc02g090360.3	1.38	Multicopper oxidase
Solyc03g122340.3	1.96	Lipoxygenase
Solyc10g054440.2	1.46	Pyridoxal-dependent decarboxylase, pyridoxal binding domain
Solyc01g057770.3	1.28	HCO3- transporter family
Solyc07g006280.3	1.42	Tetraspanin family
Solyc02g064680.3	1.87	E1-E2 ATPase

Down-regulated		
Solyc04g014280.1	-1.77	no_annotation
Solyc05g054850.1	-1.59	no_annotation
Solyc02g078250.3	-1.21	Domain of unknown function (DUF588)
Solyc11g067180.2	-1.20	Male sterility protein
Solyc02g067050.3	-2.31	Uncharacterized ACR, COG1678
Solyc06g006020.2	-1.31	Leucine Rich Repeat
Solyc07g054720.2	-2.24	Potato type II proteinase inhibitor family
Solyc02g071560.3	-1.53	Subtilase family
Solyc03g045100.1	-1.21	no_annotation
Solyc04g072280.3	-2.95	Multicopper oxidase
Solyc06g064720.1	-1.24	NB-ARC domain
Solyc07g007930.3	-1.24	Raffinose synthase or seed imbibition protein Sip1
Solyc08g077370.3	-1.14	Purine nucleobase transmembrane transport
Solyc11g006450.2	-1.22	Ring finger domain
Solyc05g007950.3	-1.65	Ribonuclease T2 family
Solyc12g006040.2	-1.06	NB-ARC domain
Solyc02g078510.3	-1.31	Transferase family
Solyc12g049320.2	-1.35	GRAS domain family
Solyc02g079940.3	-1.05	Protein of unknown function (DUF1218)
Solyc09g092605.1	-1.48	Cytochrome P450
Solyc05g054650.1	-1.24	C2H2-type zinc finger
Solyc12g006225.1	-1.52	no_annotation
Solyc01g081210.3	-1.29	no_annotation
Solyc01g095080.3	-1.50	Aminotransferase class I and II
Solyc05g051860.3	-1.33	zinc-finger of the FCS-type, C2-C2
Solyc07g053225.1	-1.46	no_annotation
Solyc12g049030.1	-3.06	Fatty acid desaturase
Solyc10g084560.2	-1.23	Peptidase inhibitor I9
Solyc07g055010.3	-1.58	Heavy-metal-associated domain
Solyc07g056080.1	-1.22	no_annotation
Solyc11g006140.1	-1.22	DnaJ domain
Solyc11g012520.1	-1.00	no_annotation
Solyc03g026360.1	-1.23	LysM domain
Solyc05g048830.3	-1.06	Myb-like DNA-binding domain
Solyc00g170200.1	-2.18	no_annotation
Solyc05g009480.1	-1.13	no_annotation
Solyc05g052680.1	-2.10	Transferase family
Solyc09g090970.3	-2.36	Pathogenesis-related protein Bet v I family
Solyc09g098080.3	-2.44	UDP-glucoronosyl and UDP-glucosyl transferase
Solyc10g055730.2	-1.06	Transferase family
Solyc12g062200.1	-3.61	no_annotation
Solyc01g044240.3	-1.29	no_annotation
Solyc02g031990.1	-1.43	VQ motif
Solyc06g065060.1	-2.25	Berberine and berberine like
Solyc08g078650.3	-1.59	Mannosyltransferase putative
Solyc12g089050.2	-1.04	Membrane bound O-acyl transferase family
Solyc01g008390.2	-1.15	Leucine rich repeat

Solyc01g009330.1	-2.15	U-box domain
Solyc04g005610.3	-1.12	No apical meristem (NAM) protein
Solyc06g072620.3	-1.08	Sugar efflux transporter for intercellular exchange
Solyc07g054790.1	-1.93	Wound-induced protein
Solyc12g009510.1	-1.13	Leucine rich repeat
Solyc05g053330.3	-1.12	Myb-like DNA-binding domain
Solyc06g062420.3	-1.62	no_annotation
Solyc00g080750.3	-1.90	Domain of unknown function (DUF4228)
Solyc03g116245.1	-1.47	Papain family cysteine protease
Solyc03g119910.3	-1.73	2OG-Fe(II) oxygenase superfamily
Solyc08g074690.3	-1.32	Protein of unknown function (DUF_B2219)
Solyc11g007500.2	-1.09	no_annotation
Solyc11g017280.2	-1.07	Leucine rich repeat
Solyc12g096960.2	-1.14	Pathogenesis-related protein Bet v I family
Solyc03g116390.3	-1.80	no_annotation
Solyc05g015850.3	-1.49	WRKY DNA -binding domain
Solyc06g008020.3	-1.15	HIT zinc finger
Solyc04g005660.3	-1.15	no_annotation
Solyc04g074020.2	-1.75	Leucine rich repeat N-terminal domain
Solyc08g080620.1	-1.34	Thaumatin family
Solyc12g096630.2	-1.04	Inosine-uridine preferring nucleoside hydrolase
Solyc02g080120.2	-1.64	2OG-Fe(II) oxygenase superfamily
Solyc00g136260.1	-1.97	Ring finger domain
Solyc04g009910.3	-1.21	Protein kinase domain
Solyc12g098880.2	-1.18	no_annotation
Solyc04g071600.3	-1.31	ABA/WDS induced protein
Solyc07g007270.3	-1.40	no_annotation
Solyc09g091670.3	-1.50	ABC-transporter extracellular N-terminal
Solyc03g119390.3	-1.55	Helix-loop-helix DNA-binding domain
Solyc07g005300.1	-1.43	no_annotation
Solyc12g009780.1	-1.20	Leucine Rich Repeat
Solyc01g097570.2	-1.04	no_annotation
Solyc02g070110.1	-2.85	Berberine and berberine like
Solyc01g109800.2	-1.14	no_annotation
Solyc05g052670.1	-2.48	Transferase family
Solyc11g069020.2	-1.50	NB-ARC domain
Solyc02g077590.1	-3.39	Homeobox domain
Solyc04g071150.3	-1.05	Cytochrome P450
Solyc04g074030.3	-1.91	Leucine rich repeat N-terminal domain
Solyc01g080800.3	-1.15	Peptidase of plants and bacteria
Solyc11g011180.2	-1.23	Leucine Rich repeat
Solyc12g008830.2	-1.45	GATA zinc finger
Solyc04g080435.1	-1.15	Myb-like DNA-binding domain
Solyc05g052890.3	-1.22	no_annotation
Solyc02g076800.1	-1.01	Domain of unknown function (DUF4228)
Solyc03g032060.1	-1.11	Ring finger domain
Solyc01g080570.3	-1.41	Inosine-uridine preferring nucleoside hydrolase
Solyc11g044910.2	-1.66	Glycosyl hydrolase family 3 N terminal domain

Solyc12g006230.2	-1.77	Ring finger domain
Solyc08g067360.3	-1.39	WRKY DNA -binding domain
Solyc12g006260.1	-1.84	no_annotation
Solyc07g045180.3	-1.23	B-box zinc finger
Solyc08g029000.3	-1.52	PLAT/LH2 domain
Solyc07g066330.3	-1.74	No apical meristem (NAM) protein
Solyc01g005040.3	-1.12	Domain of unknown function (DUF588)
Solyc06g063060.3	-1.10	Dormancy/auxin associated protein
Solyc05g012430.1	-1.71	Leucine rich repeat
Solyc12g009450.2	-1.23	NB-ARC domain
Solyc03g034000.3	-1.53	Helix-loop-helix DNA-binding domain
Solyc04g076220.3	-1.31	Domain of unknown function (DUF296)
Solyc05g055540.2	-1.15	Major Facilitator Superfamily
Solyc07g054760.1	-1.78	Wound-induced protein
Solyc09g090210.3	-1.10	Protein tyrosine kinase
Solyc08g082680.3	-1.00	Ring finger domain
Solyc07g054780.1	-1.77	Wound-induced protein
Solyc01g095690.2	-1.04	PPR repeat
Solyc08g079900.3	-1.87	Peptidase inhibitor I9
Solyc05g051200.1	-1.42	AP2 domain
Solyc07g006770.2	-1.20	TMEM154 protein family
Solyc07g052790.2	-1.00	NB-ARC domain
Solyc10g076550.1	-1.85	Protein kinase domain
Solyc04g025530.3	-1.55	Pyridoxal-dependent decarboxylase conserved domain
Solyc04g074050.3	-1.76	Protein kinase domain
Solyc01g091030.3	-1.02	Auxin responsive protein
Solyc02g085910.3	-3.04	Protein of unknown function DUF260
Solyc08g067970.3	-1.11	zinc-finger of the FCS-type, C2-C2
Solyc06g061240.3	-1.08	PLATZ transcription factor
Solyc01g008620.3	-2.50	Glycosyl hydrolases family 17
Solyc01g067020.3	-1.17	Protein kinase domain
Solyc03g006550.3	-1.06	Terpene synthase, N-terminal domain
Solyc12g089190.1	-1.27	Myb-like DNA-binding domain
Solyc09g092260.3	-2.10	DnaJ domain
Solyc12g008800.2	-1.42	Myb-like DNA-binding domain
Solyc08g080670.1	-1.49	Thaumatin family
Solyc01g091700.3	-1.17	no_annotation
Solyc03g096540.3	-2.21	PLAT/LH2 domain
Solyc04g007000.1	-1.80	AP2 domain
Solyc05g006340.3	-1.16	WD domain, G-beta repeat
Solyc07g042510.3	-1.47	Inhibitor of apoptosis-promoting Bax1
Solyc09g075920.1	-1.10	D-mannose binding lectin
Solyc07g054580.3	-1.07	GH3 auxin-responsive promoter
Solyc08g016270.2	-1.13	Leucine rich repeat
Solyc12g088840.1	-1.03	EF-hand domain
Solyc06g073165.1	-1.05	BURP domain
Solyc04g074000.3	-1.64	Protein tyrosine kinase
Solyc12g096570.1	-2.53	no_annotation

Solyc04g077860.3	-1.06	Alpha/beta hydrolase family
Solyc08g077730.3	-1.22	MORN repeat
Solyc03g096550.3	-1.82	PLAT/LH2 domain
Solyc05g053610.2	-1.27	ABC-2 type transporter
Solyc02g037495.1	-1.08	AMP-binding enzyme C-terminal domain
Solyc03g098740.1	-1.74	Trypsin and protease inhibitor
Solyc09g008830.3	-1.48	no_annotation
Solyc04g011750.3	-2.28	no_annotation
Solyc03g043740.3	-1.24	no_annotation
Solyc03g031890.3	-1.43	no_annotation
Solyc06g051680.1	-1.30	Protein of unknown function (DUF1313)
Solyc04g054260.3	-1.30	Cytochrome P450
Solyc10g083290.4	-1.24	Glycosyl hydrolases family 32 N-terminal domain
Solyc01g097240.3	-1.26	Barwin family
Solyc03g025670.3	-1.93	PAR1 protein
Solyc08g068870.3	-1.64	Xylanase inhibitor N-terminal
Solyc01g009430.3	-1.89	no_annotation
Solyc02g062140.2	-1.03	Armadillo/beta-catenin-like repeat
Solyc04g039670.3	-6.50	ATP-grasp domain
Solyc06g068500.3	-1.97	DnaJ domain
Solyc07g042190.3	-1.20	zinc-finger of the FCS-type, C2-C2
Solyc08g065940.3	-1.08	Zinc finger C-x8-C-x5-C-x3-H type (and similar)
Solyc04g009900.3	-1.27	Protein kinase domain
Solyc07g054730.1	-1.31	Wound-induced protein
Solyc09g010000.3	-1.00	2OG-Fe(II) oxygenase superfamily
Solyc01g005470.3	-1.08	PLAC8 family
Solyc08g079870.3	-1.05	PA domain
Solyc00g174340.2	-2.89	Cysteine-rich secretory protein family
Solyc02g077040.4	-1.27	Papain family cysteine protease
Solyc01g095700.3	-1.25	Polyketide cyclase / dehydrase and lipid transport
Solyc01g087800.2	-1.03	Peptidase inhibitor I9
Solyc02g082920.3	-1.11	Chitinase class I
Solyc12g009560.2	-1.14	F-box domain
Solyc10g080610.1	-1.39	Kelch motif
Solyc08g078950.3	-1.06	POT family
Solyc01g108910.3	-1.06	no_annotation
